# Unveiling the Redox Control of Plant Reproductive Development during Abiotic Stress

**DOI:** 10.3389/fpls.2016.00700

**Published:** 2016-06-16

**Authors:** Gaurav Zinta, Asif Khan, Hamada AbdElgawad, Vipasha Verma, Ashish Kumar Srivastava

**Affiliations:** ^1^Centre of Excellence Plant and Vegetation Ecology, Department of Biology, University of AntwerpAntwerp, Belgium; ^2^Integrated Molecular Plant Physiology Research, Department of Biology, University of AntwerpAntwerp, Belgium; ^3^Research Group Germline Biology, Centre for Organismal Studies Heidelberg, University of HeidelbergHeidelberg, Germany; ^4^Department of Botany, Faculty of Science, University of Beni-SuefBeni-Suef, Egypt; ^5^Department of Biotechnology, Dr Y S Parmar University of Horticulture and ForestrySolan, India; ^6^Nuclear Agriculture and Biotechnology Division, Bhabha Atomic Research CentreMumbai, India

**Keywords:** oxidative stress, antioxidants, sexual reproduction, anther, gynoecium, pollen

## Abstract

Plants being sessile in nature are often challenged to various abiotic stresses including temperature fluctuations, water supply, salinity, and nutrient availability. Exposure of plants to such environmental perturbations result in the formation of reactive oxygen species (ROS) in cells. To scavenge ROS, enzymatic and molecular antioxidants are produced at a cellular level. ROS act as a signaling entity at lower concentrations maintaining normal growth and development, but if their levels increase beyond certain threshold, they produce toxic effects in plants. Some developmental stages, such as development of reproductive organs are more sensitive to abiotic stress than other stages of growth. As success of plant reproductive development is directly correlated with grain yield, stresses coinciding with reproductive phase results in the higher yield losses. In this article, we summarize the redox control of plant reproductive development, and elaborate how redox homeostasis is compromised during abiotic stress exposure. We highlight why more emphasis should be given to understand redox control of plant reproductive organ development during abiotic stress exposure96to engineer crops with better crop yield. We specifically discuss the role of ROS as a signaling molecule and its cross-talk with other signaling molecules such as hormones and sugars.

## Introduction

The entire life cycle of flowering plants is a succession of distinct growth phases, where plants depict various developmental stages. The growth phase between seed germination and vegetative maturity is termed as “vegetative-phase”; and the subsequent phase including formation of reproductive organs, sexual reproduction, and seed set is termed as “reproductive-phase.” Plant performance in these growth phases, and transition from vegetative to reproductive phase is under tight control of genetic network (Huijser and Schmid, 2011). Moreover, signaling mediators such as reactive oxygen species (ROS), reactive nitrogen species (RNS), calcium, and phytohormones play crucial roles in integrating information from various endogenous and environmental cues, thus regulating plant growth and developmental transitions (Kocsy et al., 2013; Considine and Foyer, 2014; Traverso et al., 2014). Of these, ROS or redox mediated signaling has recently emerged as a core signaling pathway that shows crosstalk with calcium (Steinhorst and Kudla, 2013; Gilroy et al., 2014) and hormone-mediated signaling (Bartoli et al., 2013; Xia et al., 2015). The redox state is a broad term often described as an integrated ratio of different redox couples present inside the cell (König et al., 2012). Each developmental stage possess a specific redox pattern, determined by the concerted action of various ROS producing [NADPH oxidase (NOX), ascorbate oxidase (AO), and alternative oxidase (AOX)] and scavenging [superoxide dismutase (SOD), catalase (CAT), peroxidase(POD), ascorbate peroxidase (APX), glutathione peroxidase (GPX), and glutathione reductase (GR)] antioxidant enzymes and antioxidant molecules like ascorbate, glutathione, and tocopherols (Considine and Foyer, 2014). In addition, redox state is regulated by various sugars and amino acids, which apart from playing role in plant metabolism are now considered an integral part of ROS scavenging machinery (Bolouri-Moghaddam et al., 2010; Hayat et al., 2012; Matros et al., 2015).

## Redox regulation of plant sexual reproduction

**Figure 1:**
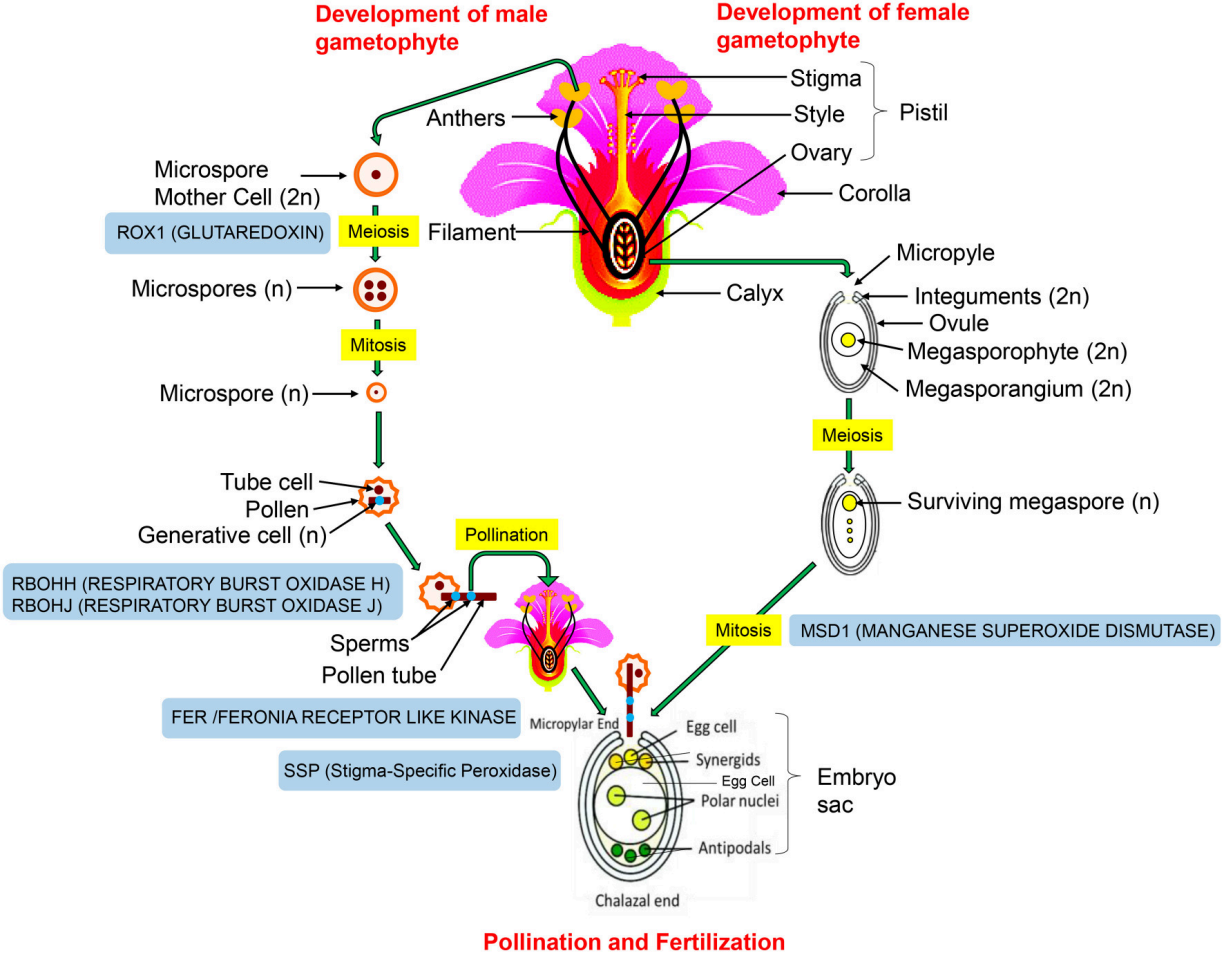
**Structure of reproductive organs and the sequence of events involved in plant sexual reproduction**. Male gametophyte (pollen grain) is comprised of a pollen tube consisting of a vegetative tube cell and two sperm cells. The female gametophyte (embryo sac) has two female gametes (egg and central cell) and accessory cells at the opposite poles. Two synergid cells adjoining the egg cell are located at micropylar entry of the ovule, while antipodal cells neighboring the central cell are present at the chalazal end. Sexual reproduction involves two major steps: pollination and fertilization. Pollination involves pollen-pistil interaction and fertilization involves fusion of meiotically generated haploid cells of a male (pollen grain) and a female gametophyte (embryo sac). During fertilization, pollen tube penetrates embryo sac at the micropyle by entering a synergid cell and delivers two sperm cells. Eventually, two gametic cell pairs of the embryo sac and two sperm cells undergo “double-fertilization.” The fertilized central cell develops into endosperm, while the fertilized egg cell gives rise to embryo. The involvement of redox proteins at different steps of sexual reproduction is highlighted in blue boxes.

In flowering plants, flower is a seat of plant sexual reproduction encapsulating both male and female reproductive organs. The sterile part of flower consists of calyx and corolla, and the fertile part consists of androecium (male) and gynoecium (female). The detailed structure of plant reproductive organs and the sequence of events leading to fertilization are described in the legend of Figure 1. Starting from male or female gametogenesis through meiosis, followed by pollen/embryo sac growth, pollen-pistil interaction, and double-fertilization—the entire process is redox regulated.

Specific ROS levels, antioxidant molecules, and enzyme activities control the individual steps of sexual reproduction (Figure 1). Arabidopsis *roxy1* mutant, deficient in CC-type GRXs (glutaredoxins: glutathione dependent oxidoreductases) displays reduced petal number and male-sterile phenotype. A deeper analysis revealed that this mutant lacks ability to enter meiotic phase (Xing and Zachgo, 2008). Furthermore, generation of reductive environment through exogenous supplementation of KI rescued the *msca1* (male sterile converted anther1: a CC-type GRX) mutant phenotype of maize, confirming that excess ROS formation was mainly responsible for male sterility (Kelliher and Walbot, 2012). Arabidopsis phytoalexin-deficient (*pad2-1*) mutant which is impaired in glutathione (GSH) synthesis shows poor pollen germination, indicating that GSH is essential for pollen development (Zechmann et al., 2011). Also, proper ROS gradient is required for pollen tube elongation. The *rbohH* and *rbohJ* (respiratory burst oxidase homolog H and J) double mutant of *Arabidopsis*, which lacks the ability of ROS burst shows severe reduction in pollen tube tip growth (Kaya et al., 2014). Similarly, ROS homeostasis is important for female gametophyte development, where mitochondrial SOD (MSD1) plays a crucial role in regulating ROS levels (Martin et al., 2013). The *mee33* (maternal effect embryo arrest 33) mutant lacking MSD1 activity shows defects in embryo sac development. The sperm cell release from pollen tube is a redox (ROS) dependent process. It is regulated through FERONIA (FER) receptor kinase which induces the production of high ROS levels, especially hydroxyl radicals, at the entrance of female gametophyte to facilitate the rupture of pollen tube in a calcium dependent manner (Duan et al., 2014). Interaction between pollen and pistil during pollination is one of a key step that determines the fate of fertilization, and decides whether seed set will happen or not. ROS (H_2_O_2_) and NO (nitric oxide) mediated redox signaling is involved in pollen-pistil interactions (Sharma and Bhatla, 2013). Before pollen-pistil interaction, higher H_2_O_2_ production and enhanced activity of a Stigma-Specific Peroxidase (SSP) was observed at the receptive papillae, while pollen still retain high NO levels (McInnis et al., 2006; Bright et al., 2009; Zafra et al., 2010). After pollen landing on stigma, during the period of pollen-pistil interaction, the levels of H_2_O_2_ and activity of SSP declines dramatically in the stigmatic papillae. Following successful fertilization, formation of an embryo, and the development of mature seed requires extensive cell division and cell expansion, and both these processes are again redox regulated, and require low molecular weight antioxidants such as ascorbate and glutathione (Cairns et al., 2006; Livanos et al., 2012; Gallie, 2013). Thus, well-coordinated changes in the redox metabolism is essential for successful plant sexual reproduction. Such concept has also been proposed for other organisms such as *Caenorhabditis elegans* (De Henau et al., 2015) and mammals (Ufer et al., 2010).

## Abiotic stress induces redox imbalance during reproductive growth

Exposure of plants to abiotic stresses such as temperature fluctuations, water supply, and salinity, result in the formation of ROS (Gill and Tuteja, 2010; Suzuki et al., 2012; Zinta et al., 2014). Although, ROS acts as a signaling molecule at lower concentrations, it's accumulation beyond threshold leads to oxidative damage of macromolecules, resulting in growth retardation (Mittler, 2002). Because the success of plant reproductive development determines grain yield, it is obvious that stress exposure during reproductive phase will reduce the crop yield (Dolferus et al., 2011; Sage et al., 2015). Therefore, it is essential to understand how stress affects redox homeostasis in the context of plant sexual reproduction.

Stress negatively affects male reproductive development in plants (De Storme and Geelen, 2014). Drought imposed to rice during anthesis resulted in generation of sterile pollen, due to higher ROS accumulation and lower expression of transcripts related to antioxidant enzymes (Selote and Khanna-Chopra, 2004; Nguyen et al., 2009). Similarly, a study performing the comparative analysis of anthers of a drought sensitive and tolerant rice lines (under drought stress) revealed sensitive line exhibiting higher malondialdehyde (MDA) content and lower activity of antioxidant enzymes (SOD, POD, and CAT) as compared to tolerant line (Fu et al., 2011). These studies indicate that stress-induced over accumulation of ROS leads to pollen abortion and programmed cell death (PCD) of microspores in developing anthers, consequently resulting in male sterility.

Similarly, the female reproductive development is impaired by abiotic stress exposure (Moss and Downey, 1971; Sun et al., 2004; Loka and Oosterhuis, 2014). Heat stress caused activation of GR activity in the heat-stressed cotton pistils (Snider et al., 2009). Thermotolerant cotton variety showed higher pre-stress activity of SOD and GR in the pistil, suggesting it as a thermotolerance mechanism specific to female reproductive organs (Snider et al., 2011). Moreover, in salt stressed *Arabidopsis* plants, genes encoding ROS detoxifying enzymes, APX, and POD, were downregulated after ovules committed to abort. These changes in gene expression coincided with the accumulation of ROS in female gametophytes (Sun et al., 2005). This may have resulted from increased ROS biosynthesis, reduced ROS scavenging capacity or both, indicating that like male gametophyte, female gametophyte is also susceptible to stress-induced ROS accumulation.

Sugars or sugar-mediated transcriptional control play an important role in ROS homeostasis in the reproductive organs during stress episode (Couée et al., 2006; Keunen et al., 2013). Reduction in starch content prior to anthesis and decline in total soluble sugar content in mature pollen grains resulted in decreased pollen viability in tomato plants exposed to high temperature (Pressman et al., 2002). Recent metabolomic and transcriptomic profiling of floral organs (anthers and pistil) of heat-tolerant (N22) and heat-sensitive (Moroberekan) rice cultivars identified modulation in sugar metabolism as a regulatory mechanism imparting heat and drought tolerance to floral organs (Li et al., 2015). Therefore, stress-induced redox imbalance due to inefficient antioxidant system or alterations in the sugar metabolism could lead to higher ROS accumulation, which results in PCD of developing microspores/megaspores, finally leading to male sterility or ovule abortion.

## Engineering redox components for improved reproductive success

A dynamic network of redox homeostasis related genes function to repair or abrogate stress-induced oxidative damage in the plant reproductive tissues (Meyer et al., 2012). Therefore, engineering redox components could be a way forward to impart stress tolerance in plants during reproductive phase.

The role of plant GRXs as master regulators of redox homeostasis during anther and gamete formation has been well-demonstrated (Kelliher and Walbot, 2012). A conserved plant specific CBSX (single cystathionine β-synthase domain) CC-type GRXs (ROXY1 and ROXY 2) genes and SQUAMOSA PROMOTER BINDING PROTEIN (SBP-box) transcription factors (target of miR156 and miR157) have been implicated in redox clean up during male reproductive development (Yoo et al., 2011). The role of these intriguing proteins was revealed in the studies using maize *msca1* mutant (Chaubal et al., 2003; Timofejeva et al., 2013). The CC-type GRXs, ROXY 1 and 2 act via interaction with basic leuine-zipper transcription factors, TGACG (TGA) motif-binding proteins TGA9 and TGA10 (Murmu et al., 2010). This association was demonstrated later in rice, where authors showed that rice MICROSPORELESS1(MIL1) gene, coding for a similar CC-type GRX, functions in the formation of surrounding somatic layer of anthers and in the transition of microsporocytes from mitosis to meiosis (Hong et al., 2012).

The role of cytosolic ascorbate peroxidase 2 (APX2) in enhanced seed production subjected to chronic heat stress was illustrated in *Arabidopsis thaliana* (Suzuki et al., 2013). Similarly, heat-stressed microspores in tomato showed upregulation of ROS-scavenging SlAPX3, safeguarding spores against toxic ROS accumulation (Frank et al., 2009). These two are relevant findings in the context of global warming, and demonstrate the protective role of APX against heat-induced oxidative damage in reproductive tissues.

One of plant's remarkable strategy to deal with stress is to escape the stressful environment via reproduction. Hence, proper timing of transition from vegetative to reproductive phase determines the success of reproduction. The OXS2 (OXIDATIVE STRESS 2) is a member of zinc-finger transcription factor family, which is involved in maintaining vegetative growth, stress tolerance, and stress-induced reproduction (Blanvillain et al., 2011). OXS2 is a stress responsive nucleo-cytoplasmic protein, shuttling between cytoplasm (no stress) to nucleus under stress conditions promoting stress resilience. OXS2 autoactivates itself, while coherently activating other floral integrator genes via direct binding to floral integrator promoter SUPPRESOR OF CONSTANS (SOC1). This auto-regulatory loop of OXS2 may constitute an altruistic response choosing between stress tolerance to stress escape via reproduction (Blanvillain et al., 2011).

Two contrasting hypothesis have been proposed to explain the effects of redox status on meiosis and germline formation. The “reductive hypothesis” proposes that reduced ROS levels are required for successful meiosis (Kelliher and Walbot, 2014). This is supported by the evidences where chemical (Kelliher and Walbot, 2012) or genetic (Zechmann et al., 2011) approaches were used to reduce ROS levels, resulting in improved reproductive success as well as crop yield. In contrast, the “oxidative hypothesis” advocates increased ROS levels as a prerequisite for initiation of meiosis and sexual reproduction (Hörandl and Hadacek, 2013). In this view, the role of a meiotic protein SPO11 has been proposed. This protein initiates generation of DSBs (double-strand DNA breaks) during meiotic recombination and have antioxidant like properties to repair oxidatively damaged DNA (Hörandl and Hadacek, 2013). Moreover, oxidative stress can deregulate the epigenetic machinery comprising of DNA methylation and small RNA-based transcriptional regulation (Schmidt et al., 2015; Zhang et al., 2015). Thus, plant sexual reproduction is a highly complicated process which requires a fine-tuning of oxidative-reductive (redox) pathways as well as epigenetic mechanisms, hence complicating the engineering of modules for enhancing stress tolerance in plants.

## Conclusions

The redox control of plant reproductive development has been the long standing dilemma of plant scientists. Understanding the reproductive cost of oxidative stress requires a targeted molecular approach for engineering transgenes responsible for stress resilience in plant's reproductive system. But our knowledge is demarcated by methods to intertwine regulatory networks underlying reproductive development. Although much of the studies now focus on *Arabidopsis* as a model organism, future research should input judicious use of major crop species ensuring yield robustness in meeting continual demands of an ever increasing human population.

## Author contributions

GZ and AK conceived the idea and outline for the prescribed article and thereafter compiled and incorporated the different sections contributed by co-authors. All the authors read and approved the final version before submission.

### Conflict of interest statement

The authors declare that the research was conducted in the absence of any commercial or financial relationships that could be construed as a potential conflict of interest.

## References

[B1] Bartoli C. G. Casalongue C. A. Simontacchi M. Marquez-Garcia B. Foyer C. H. (2013). Interactions between hormone and redox signalling pathways in the control of growth and cross tolerance to stress. Environ. Exp. Bot. 94, 73–88. 10.1016/j.envexpbot.2012.05.003

[B2] Blanvillain R. Wei S. Wei P. Kim J. H. Ow D. W. (2011). Stress tolerance to stress escape in plants: role of the OXS2 zinc-finger transcription factor family. EMBO J. 30, 3812–3822. 10.1038/emboj.2011.270 21829164 PMC3173794

[B3] Bolouri-Moghaddam M. R. Le Roy K. Xiang L. Rolland F. Van Den Ende W. (2010). Sugar signalling and antioxidant network connections in plant cells. FEBS J. 277, 2022–2037. 10.1111/j.1742-4658.2010.07633.x 20412056

[B4] Bright J. Hiscock S. J. James P. E. Hancock J. T. (2009). Pollen generates nitric oxide and nitrite: a possible link to pollen-induced allergic responses. Plant Physiol. Biochem. 47, 49–55. 10.1016/j.plaphy.2008.09.005 18964065

[B5] Cairns N. G. Pasternak M. Wachter A. Cobbett C. S. Meyer A. J. (2006). Maturation of Arabidopsis seeds is dependent on glutathione biosynthesis within the embryo. Plant Physiol. 141, 446–455. 10.1104/pp.106.077982 16531482 PMC1475471

[B6] Chaubal R. Anderson J. R. Trimnell M. R. Fox T. W. Albertsen M. C. Bedinger P. (2003). The transformation of anthers in the msca1 mutant of maize. Planta 216, 778–788. 10.1007/s00425-002-0929-8 12624765

[B7] Considine M. J. Foyer C. H. (2014). Redox regulation of plant development. Antioxid. Redox Signal. 21, 1305–1326. 10.1089/ars.2013.5665 24180689 PMC4158970

[B8] Couée I. Sulmon C. Gouesbet G. El Amrani A. (2006). Involvement of soluble sugars in reactive oxygen species balance and responses to oxidative stress in plants. J. Exp. Bot. 57, 449–459. 10.1093/jxb/erj027 16397003

[B9] De Henau S. Tilleman L. Vangheel M. Luyckx E. Trashin S. Pauwels M.. (2015). A redox signalling globin is essential for reproduction in *Caenorhabditis elegans*. Nat. Commun. 6, 8782. 10.1038/ncomms9782 26621324 PMC4686822

[B10] De Storme N. Geelen D. (2014). The impact of environmental stress on male reproductive development in plants: biological processes and molecular mechanisms. Plant Cell Environ. 37, 1–18. 10.1111/pce.12142 23731015 PMC4280902

[B11] Dolferus R. Ji X. Richards R. A. (2011). Abiotic stress and control of grain number in cereals. Plant Sci. 181, 331–341. 10.1016/j.plantsci.2011.05.015 21889038

[B12] Duan Q. Kita D. Johnson E. A. Aggarwal M. Gates L. Wu H. M.. (2014). Reactive oxygen species mediate pollen tube rupture to release sperm for fertilization in Arabidopsis. Nat. Commun. 5, 3129. 10.1038/ncomms4129 24451849

[B13] Frank G. Pressman E. Ophir R. Althan L. Shaked R. Freedman M. (2009). Transcriptional profiling of maturing tomato (*Solanum lycopersicum* L.) microspores reveals the involvement of heat shock proteins, ROS scavengers, hormones, and sugars in the heat stress response. J. Exp. Bot. 60, 3891–3908. 10.1093/jxb/erp234 19628571 PMC2736902

[B14] Fu G.-F. Jian S. Xiong J. Li Y.-R. Chen H.-Z. Le M.-K. (2011). Changes of oxidative stress and soluble sugar in anthers involve in rice pollen abortion under drought stress. Agric. Sci. China 10, 1016–1025. 10.1016/S1671-2927(11)60089-8

[B15] Gallie D. R. (2013). L-ascorbic Acid: a multifunctional molecule supporting plant growth and development. Scientifica 2013:795964. 10.1155/2013/795964 24278786 PMC3820358

[B16] Gill S. S. Tuteja N. (2010). Reactive oxygen species and antioxidant machinery in abiotic stress tolerance in crop plants. Plant Physiol. Biochem. 48, 909–930. 10.1016/j.plaphy.2010.08.016 20870416

[B17] Gilroy S. Suzuki N. Miller G. Choi W. G. Toyota M. Devireddy A. R.. (2014). A tidal wave of signals: calcium and ROS at the forefront of rapid systemic signaling. Trends Plant Sci. 19, 623–630. 10.1016/j.tplants.2014.06.013 25088679

[B18] Hayat S. Hayat Q. Alyemeni M. N. Wani A. S. Pichtel J. Ahmad A. (2012). Role of proline under changing environments: a review. Plant Signal. Behav. 7, 1456–1466. 10.4161/psb.21949 22951402 PMC3548871

[B19] Hong L. Tang D. Shen Y. Hu Q. Wang K. Li M.. (2012). MIL2 (MICROSPORELESS2) regulates early cell differentiation in the rice anther. New Phytol. 196, 402–413. 10.1111/j.1469-8137.2012.04270.x 22913653

[B20] Hörandl E. Hadacek F. (2013). The oxidative damage initiation hypothesis for meiosis. Plant Reprod. 26, 351–367. 10.1007/s00497-013-0234-7 23995700 PMC3825497

[B21] Huijser P. Schmid M. (2011). The control of developmental phase transitions in plants. Development 138, 4117–4129. 10.1242/dev.063511 21896627

[B22] Kaya H. Nakajima R. Iwano M. Kanaoka M. M. Kimura S. Takeda S.. (2014). Ca2+-activated reactive oxygen species production by Arabidopsis RbohH and RbohJ is essential for proper pollen tube tip growth. Plant Cell 26, 1069–1080. 10.1105/tpc.113.120642 24610725 PMC4001369

[B23] Kelliher T. Walbot V. (2012). Hypoxia triggers meiotic fate acquisition in maize. Science 337, 345–348. 10.1126/science.1220080 22822150 PMC4101383

[B24] Kelliher T. Walbot V. (2014). Maize germinal cell initials accommodate hypoxia and precociously express meiotic genes. Plant J. 77, 639–652. 10.1111/tpj.12414 24387628 PMC3928636

[B25] Keunen E. Peshev D. Vangronsveld J. Van Den Ende W. Cuypers A. (2013). Plant sugars are crucial players in the oxidative challenge during abiotic stress: extending the traditional concept. Plant Cell Environ. 36, 1242–1255. 10.1111/pce.12061 23305614

[B26] Kocsy G. Tari I. Vanková R. Zechmann B. Gulyás Z. Poór P.. (2013). Redox control of plant growth and development. Plant Sci. 211, 77–91. 10.1016/j.plantsci.2013.07.004 23987814

[B27] König J. Muthuramalingam M. Dietz K. J. (2012). Mechanisms and dynamics in the thiol/disulfide redox regulatory network: transmitters, sensors and targets. Curr. Opin. Plant Biol. 15, 261–268. 10.1016/j.pbi.2011.12.002 22226570

[B28] Li X. Lawas L. M. Malo R. Glaubitz U. Erban A. Mauleon R.. (2015). Metabolic and transcriptomic signatures of rice floral organs reveal sugar starvation as a factor in reproductive failure under heat and drought stress. Plant Cell Environ. 38, 2171–2192. 10.1111/pce.12545 25828772

[B29] Livanos P. Apostolakos P. Galatis B. (2012). Plant cell division: ROS homeostasis is required. Plant Signal. Behav. 7, 771–778. 10.4161/psb.20530 22751303 PMC3583961

[B30] Loka D. Oosterhuis D. (2014). Water-deficit stress effects on pistil biochemistry and leaf physiology in cotton (*Gossypium hirsutum*, L.). South Afr. J. Bot. 93, 131–136. 10.1016/j.sajb.2014.03.019

[B31] Martin M. V. Fiol D. F. Sundaresan V. Zabaleta E. J. Pagnussat G. C. (2013). Oiwa, a female gametophytic mutant impaired in a mitochondrial manganese-superoxide dismutase, reveals crucial roles for reactive oxygen species during embryo sac development and fertilization in Arabidopsis. Plant Cell 25, 1573–1591. 10.1105/tpc.113.109306 23653473 PMC3694693

[B32] Matros A. Peshev D. Peukert M. Mock H. P. Van den Ende W. (2015). Sugars as hydroxyl radical scavengers: proof-of-concept by studying the fate of sucralose in Arabidopsis. Plant J. 82, 822–839. 10.1111/tpj.12853 25891826

[B33] McInnis S. M. Desikan R. Hancock J. T. Hiscock S. J. (2006). Production of reactive oxygen species and reactive nitrogen species by angiosperm stigmas and pollen: potential signalling crosstalk? New Phytol. 172, 221–228. 10.1111/j.1469-8137.2006.01875.x 16995910

[B34] Meyer Y. Belin C. Delorme-Hinoux V. Reichheld J.-P. Riondet C. (2012). Thioredoxin and glutaredoxin systems in plants: molecular mechanisms, crosstalks, and functional significance. Antioxid. Redox Signal. 17, 1124–1160. 10.1089/ars.2011.4327 22531002

[B35] Mittler R. (2002). Oxidative stress, antioxidants and stress tolerance. Trends Plant Sci. 7, 405–410. 10.1016/S1360-1385(02)02312-9 12234732

[B36] Moss G. Downey L. (1971). Influence of drought stress on female gametophyte development in corn (*Zea mays* L.) and subsequent grain yield. Crop Sci. 11, 368–372. 10.2135/cropsci1971.0011183X001100030017x

[B37] Murmu J. Bush M. J. Delong C. Li S. Xu M. Khan M.. (2010). Arabidopsis basic leucine-zipper transcription factors TGA9 and TGA10 interact with floral glutaredoxins ROXY1 and ROXY2 and are redundantly required for anther development. Plant Physiol. 154, 1492–1504. 10.1104/pp.110.159111 20805327 PMC2971623

[B38] Nguyen G. Hailstones D. Wilkes M. Sutton B. (2009). Drought-induced oxidative conditions in rice anthers leading to a programmed cell death and pollen abortion^*^. J. Agron. Crop Sci. 195, 157–164. 10.1111/j.1439-037X.2008.00357.x

[B39] Pressman E. Peet M. M. Pharr D. M. (2002). The effect of heat stress on tomato pollen characteristics is associated with changes in carbohydrate concentration in the developing anthers. Ann. Bot. 90, 631–636. 10.1093/aob/mcf240 12466104 PMC4240456

[B40] Sage T. L. Bagha S. Lundsgaard-Nielsen V. Branch H. A. Sultmanis S. Sage R. F. (2015). The effect of high temperature stress on male and female reproduction in plants. Field Crops Res. 182, 30–42. 10.1016/j.fcr.2015.06.011

[B41] Schmidt A. Schmid M. W. Grossniklaus U. (2015). Plant germline formation: commonconcepts and developmental flexibility in sexual and asexual reproduction. Development 142, 229–241. 10.1242/dev.102103 25564620

[B42] Selote D. S. Khanna-Chopra R. (2004). Drought-induced spikelet sterility is associated with an inefficient antioxidant defence in rice panicles. Physiol. Plant. 121, 462–471. 10.1111/j.1399-3054.2004.00341.x

[B43] Sharma B. Bhatla S. (2013). Accumulation and scavenging of reactive oxygen species and nitric oxide correlate with stigma maturation and pollen–stigma interaction in sunflower. Acta Physiol. Plant. 35, 2777–2787. 10.1007/s11738-013-1310-1

[B44] Snider J. L. Oosterhuis D. M. Skulman B. W. Kawakami E. M. (2009). Heat stress-induced limitations to reproductive success in *Gossypium hirsutum*. Physiol. Plant. 137, 125–138. 10.1111/j.1399-3054.2009.01266.x 19656331

[B45] Snider J. Oosterhuis D. Kawakami E. (2011). Mechanisms of reproductive thermotolerance in *Gossypium hirsutum*: the effect of genotype and exogenous calcium application. J. Agron. Crop Sci. 197, 228–236. 10.1111/j.1439-037X.2010.00457.x

[B46] Steinhorst L. Kudla J. (2013). Calcium and reactive oxygen species rule the waves of signaling. Plant Physiol. 163, 471–485. 10.1104/pp.113.222950 23898042 PMC3793029

[B47] Sun K. Cui Y. Hauser B. A. (2005). Environmental stress alters genes expression and induces ovule abortion: reactive oxygen species appear as ovules commit to abort. Planta 222, 632–642. 10.1007/s00425-005-0010-5 16133218

[B48] Sun K. Hunt K. Hauser B. A. (2004). Ovule abortion in Arabidopsis triggered by stress. Plant Physiol. 135, 2358–2367. 10.1104/pp.104.043091 15299130 PMC520803

[B49] Suzuki N. Koussevitzky S. Mittler R. Miller G. (2012). ROS and redox signalling in the response of plants to abiotic stress. Plant Cell Environ. 35, 259–270. 10.1111/j.1365-3040.2011.02336.x 21486305

[B50] Suzuki N. Miller G. Sejima H. Harper J. Mittler R. (2013). Enhanced seed production under prolonged heat stress conditions in *Arabidopsis thaliana* plants deficient in cytosolic ascorbate peroxidase 2. J. Exp. Bot. 64, 253–263. 10.1093/jxb/ers335 23183257 PMC3528037

[B51] Timofejeva L. Skibbe D. S. Lee S. Golubovskaya I. Wang R. Harper L.. (2013). Cytological characterization and allelism testing of anther developmental mutants identified in a screen of maize male sterile lines. G3 (Bethesda) 3, 231–249. 10.1534/g3.112.004465 23390600 PMC3564984

[B52] Traverso J. A. Pulido A. Rodríguez-García M. I. Alché J. D. (2014). Thiol-based redox regulation in sexual plant reproduction: new insights and perspectives. Front Plant Sci. 4:465. 10.3389/fpls.2013.00465 24294217 PMC3827552

[B53] Ufer C. Wang C. C. Borchert A. Heydeck D. Kuhn H. (2010). Redox control in mammalian embryo development. Antioxid. Redox Signal. 13, 833–875. 10.1089/ars.2009.3044 20367257

[B54] Xia X. J. Zhou Y. H. Shi K. Zhou J. Foyer C. H. Yu J. Q. (2015). Interplay between reactive oxygen species and hormones in the control of plant development and stress tolerance. J. Exp. Bot. 66, 2839–2856. 10.1093/jxb/erv089 25788732

[B55] Xing S. Zachgo S. (2008). ROXY1 and ROXY2, two Arabidopsis glutaredoxin genes, are required for anther development. Plant J. 53, 790–801. 10.1111/j.1365-313X.2007.03375.x 18036205

[B56] Yoo K. S. Ok S. H. Jeong B.-C. Jung K. W. Cui M. H. Hyoung S.. (2011). Single cystathionine β-synthase domain–containing proteins modulate development by regulating the thioredoxin system in Arabidopsis. Plant Cell 23, 3577–3594. 10.1105/tpc.111.089847 22021414 PMC3229136

[B57] Zafra A. Rodríguez-García M. I. Alché J. D. (2010). Cellular localization of ROS and NO in olive reproductive tissues during flower development. BMC Plant Biol. 10:36. 10.1186/1471-2229-10-36 20181244 PMC2838403

[B58] Zechmann B. Koffler B. E. Russell S. D. (2011). Glutathione synthesis is essential for pollen germination *in vitro*. BMC Plant Biol. 11:54. 10.1186/1471-2229-11-54 21439079 PMC3078877

[B59] Zhang H. Xia R. Meyers B. C. Walbot V. (2015). Evolution, functions, and mysteries of plant ARGONAUTE proteins. Curr. Opin. Plant Biol. 27, 84–90. 10.1016/j.pbi.2015.06.011 26190741

[B60] Zinta G. Abdelgawad H. Domagalska M. A. Vergauwen L. Knapen D. Nijs I.. (2014). Physiological, biochemical, and genome-wide transcriptional analysis reveals that elevated CO2 mitigates the impact of combined heat wave and drought stress in *Arabidopsis thaliana* at multiple organizational levels. Glob. Chang. Biol. 20, 3670–3685. 10.1111/gcb.12626 24802996

